# The Molecular Genetics of Autism Spectrum Disorders: Genomic Mechanisms, Neuroimmunopathology, and Clinical Implications

**DOI:** 10.1155/2011/398636

**Published:** 2011-05-17

**Authors:** Daniel J. Guerra

**Affiliations:** Veterinary and Comparative Anatomy Physiology and Pathology, College of Veterinary Medicine, Washington State University, Pullman, WA 99164, USA

## Abstract

Autism spectrum disorders (ASDs) have become increasingly common in recent years. The discovery of single-nucleotide polymorphisms and accompanying copy number variations within the genome has increased our understanding of the architecture of the disease. These genetic and genomic alterations coupled with epigenetic phenomena have pointed to a neuroimmunopathological mechanism for ASD. Model animal studies, developmental biology, and affective neuroscience laid a foundation for dissecting the neural pathways impacted by these disease-generating mechanisms. The goal of current autism research is directed toward a systems biological approach to find the most basic genetic and environmental causes to this severe developmental disease. It is hoped that future genomic and neuroimmunological research will be directed toward finding the road toward prevention, treatment, and cure of ASD.

## 1. Introduction

Autism spectrum disorders (ASDs) are a complex set of human neuropsychiatric diseases that present in very early childhood and can persist throughout life. While many years of research publications were unable to describe a direct causal relationship or pattern for acquiring these disorders, it is well established that ASD is one of the most heritable and therefore genome-based human diseases. Besides a strong genetic component (monozygotic twins have ca. 90% chance of sharing the disease while dizygotic twins have only a 5–10% comorbidity), no single gene has emerged as a specifically linked cause for autism. Recently, many candidate genes have been linked to ASD, but no single allele is common for all who share the disease. Besides this strong yet elusive genetic determinant, the environment also appears to play a role in ASD. Many conflicting theories have been presented to explain the environmental components of the disease including aspects of nutrition, economic status, vaccination, and general health care as well as environmental pollutants and family life. None of these environmental parameters have been shown to be commonly linked to ASD. Indeed the anomalies of ASD include the high incidence in middle and upper middle class young children as opposed to underprivileged kids living in poverty [1]. There is one rather mysterious risk factor for ASD and that is the fact that male children are 4-5 times more likely to have ASD than female children. There is one notable exception to this rule in the female-dominated Rhett's syndrome, a form of autism found in young girls who carry a lesion in the MECP-2 gene which is involved in maintaining the methylation pattern of sexually imprinted or environmentally induced methylation patterns of cytosine residues in promoter regions of specific nuclear genes. Besides this and a few other very rare ASD, most often the disease presents 4-5 times more often in infant male children.

ASD is characterized by diverse cellular and anatomical processes that appear during early stages of human development. These include aspects of neurogenesis, neuronal migration, maturation, differentiation, and degeneration. There are also striking examples of overexpansion of certain regions of the prefrontal cortex and cerebellum in general, as well as multiple sites of dysregulation in both the innate and acquired immune response.

This paper will examine some of the known genes that cause autism plus an examination of the mechanisms for ASD-linked genomic alterations. There are implications for epigenetic and chromatin remodeling events at the single gene level but also in coordinated larger genetic networks. Some of these phenomena involve inherited and sex-linked mutations while others appear to arise *de novo* in the population. These are characterized by single-nucleotide polymorphisms and most importantly by copy number variations that result from genome-wide chromosomal abnormalities including large deletions and duplications. The immune system appears to play a major role in this disease. A systems biological approach to understanding ASD is warranted. We will conclude with a brief synopsis of the role of animal models of autism and how understanding of the underlying brain mechanisms could lead to prevention and new treatment options.

## 2. ASD Genes

The following is a description of a set of autism-linked genes. While this is not an exhaustive examination, it should provide a window to the complexity and divergent spectrum of biological processes that appear to be correlated to ASD. For purposes of keeping this paper succinct, only those genes which have been directly reported in ASD patients will be considered.

Deleted in autism (DIA-1) appeared as a deletion in a much larger deleted region of chromosome 3q24. This is a homozygous rare deletion that was first characterized in a consanguinous pedigree where ASD had been reported [1]. This identified 886-kbp homozygous deletion is hemizygotic in the patient's parents. However, DIA-1 was not screened positive in over 2000 other ASD patients nor had it surfaced in the autism genetic resource exchange (AGRE) repository [AGRE, http://www.agre.org/]. The DIA-1 deletion maps to a protein product of the Golgi. This exemplifies the lack of common causality among ASD populations. 

DIA-1 or GoPro49 directly links to the processing center of the Golgi apparatus where posttranslation glycosylation and the secretory pathway are found. GoPro49 is specifically expressed in the multipotent cells of the mesenchyme during tissue differentiation [2]. The mesenchymal cell lineage includes adipocytes, chondrocytes, and osteoblast lineages. GoPro49 is typically expressed in vertebrae and in the craniofacial nasal septum and dental follicle. In the vertebrae, GoPro49 protein levels decrease before final chondrocyte differentiation, while in the craniofacial area, expression continues postnatally. Of particular significance, the DIA-1, being a component of the mesenchymal cell lineage, is involved in the production of lymphoid tissues [2]. We will discuss the implications of DIA-1 in networking of autism-linked genes and physiological implications in a later section of this paper.

Both neuroligins and neurexins have been implicated in ASD. Neuroligins are proteins that serve as postsynaptic cell-adhesion molecules. They are colocalized with *β*-neurexins, proteins which are bound to the presynaptic membrane and which together create a cell-adhesion complex for neural synaptic interactions [3]. Neuroligins are characterized by their primary and secondary structure which include an extensive extracellular N-terminal domain somewhat homologous to the *α*/*β*-hydrolase fold domain of acetylcholinesterase. Neuroligins also possess an extensive *O*-linked oligosaccharide binding domain, as well as a transmembrane region, and ultimately terminate in a short cytoplasmic carboxy terminus. The regulation and specificity of neuroligin binding to *β*-neurexins involves alternative splicing and subunit rearrangement. Since neuroligins are found postsynaptically and neurexins organized presynaptically, a putative transsynaptic complex with functional and structural properties is found as a synaptic architectural component. *β*-neurexins serve as neuroligin ligands, and their interaction leads to synaptogenesis [4]. Since *β*-neurexins appear to cause synaptogenesis, it is likely the corruption of this interaction which results in an interruption of action potential that may account for some behavioral patterns observed in ASD. For example, in a rodent model, recent work has demonstrated that certain behavioral patterns are associated with neurexin expression. One such paper [2] showed that neurexin-1*α*-deficient mice, but not controls, demonstrated an increase in grooming and yet a distinct inability to build nests. These same neurexin-deficient mice did, however, have an enhanced capacity for motor learning. Discretely, mice deficient in neurexin-1*α* did not appear hindered in social interaction. This knockout mouse study suggests that a deficiency in neurexin-1*α* expression produces a specific “neural phenotype” which is similar to humans with the same genetic deficiency. This points to the complex nature of ASD and the underlying spectrum of disease and symptom-causing genes. 

Neurexins exist as multiple isoforms. Isoform expression of neurexins has unique molecular biological roles. It has been established that neurexins and neuroligins play a role in functional synaptogenesis in the CNS. In particular, both cholinergic and glutaminergic [5] neuronal synaptogenesis are controlled by neurexin isoform expression. For example, the exact spacing and geometry of nicotinic acetylcholine receptors (AChRs) are essential criteria for functionality. Recently, it was shown that certain AChRs require require neurexin-1*β* functioning as a synaptic cell adhesion protein [6]. Given an important theory about the ratio of excitory versus inhibitory neuronal activity in ASD, these data point to the neurexin and neuroligin gene family as significant components of this “signal to noise” hypothesis [7]. Neurexins and neuroligins are therefore important autism-linked genes as they are associated with synapse formation and functional physiology [8]. Another interesting point about these neural proteins is their necessary role in muscle and endothelial cell function [4]. Given that ASD is a cognitive, behavioral, and vascular phenomenon, it is not surprising that certain genes play a role in more than one component of disease presentation. As new research indicates, the neurexin/neuroligin family of cell adhesion molecules and their activity in synaptogenesis continues to grow as new protein ligands (e.g., leucine-rich repeat transmembrane proteins, LRRTMs) become recognized as being coordinated by neurexin and neuroligin gene expression [9].

Protocadherins are calcium-dependent cell adhesion molecules which, upon mutation, cause some forms of autism [1]. In particular, large deletions which accompany loss of PCDH 10 have been linked to ASD. While the majority of ASD is young male specific, certain forms are found in young girls. Besides the Rett syndrome (see below), a comorbid association of epilepsy and mental retardation (EFMR) appears to be an X chromosome-linked ASD which presents in heterozygous females but not in comparable hemizygous males. EFMR is associated with mutations in the protocadherin 19 (*PCDH19*) gene [10]. As with neurexins and neuroligins, there are multiple roles for PCDH isoforms in human biology. PCDH-mediated cell-cell adhesion is essential for neural tube formation [11]. These findings coupled with deletion studies in PCDH genes and association with ASD demonstrate the potential for pleiotropic and complex interactions. 

Another important gene that has been implicated in ASD includes UBE3A [1]. Mutated forms of UBE3A (a ubiquitin 3 CoA ligase which is a member of a larger family of ubiquitin–proteasomal degradation pathway genes) are found in ASD. Effects of gene deletion and amplification on the expression of specific ASD genes are common in the literature, and the mechanisms for these genome-wide alterations are a key component. *UBE3A* found on chromosome 15q11–q13 is upregulated in ASD. Furthermore, an increase in UBE3A levels may induce regulatory changes in protein ubiquitination which may occur upon gene duplication or at the level of protein amino acid substitutions and deletions. [12]. An ASD-like associated neuropsychiatric condition known as Angleman's syndrome is characterized with similar behavioral presentation (cognitive, behavioral, and motor dysfunction) as some autisms and significantly is linked to alterations of the UBE3A gene. The functional elimination of maternal, but not paternal, *UBE3A* specifically causes Angelman syndrome. The striking correlation that maternal chromosome15-associated *UBE3A* duplications cause autism suggests that proteasomal degradation pathways in the brain are related to the disease. 

Indeed, The *UBE3A* gene product, E6-AP, is both a traditional E3 ligase as well as a fully functional transcriptional coactivator. The maternally inherited E6-AP appears in the nucleus in both presynaptic and postsynaptic neurons of the hippocampus. To demonstrate how lack of expression of E6-AP is tissue and function specific, in this case, the number of cerebellar Purkinje cells is not altered. Indeed, lack of expression does not alter dendritic branching either. However, loss of function does disrupt dendritic spine development and the morphology of cortical and hippocampal pyramidal neurons. Thus, it appears that the UBE3A gene product, E6-AP, specifically controls dendritic morphology and function in discrete regions of the brain and that loss of this protein is directly linked to ASD [13]. A related ubiquitin ligase/proteasomal gene product, the Ring finger 8 (RNF8) protein, is also implicated in some forms of autism. This protein associates with the retinoic acid nuclear receptor complex (RXR-*α*) as well as the ubiquitin conjugating enzyme complex, thus making it both a transcriptional coregulator as well as a vital component of protein degradation [14]. 

A closely related autistic disorder, the Smith-Magenis syndrome (SMS), is caused by a large genomic deletion in chromosome 17p. SMA presents with severe cognitive, physical, and behavioral dysfunction. A reciprocal duplication of this genomic region on chromosome 17 is correlated with another ASD neuropsychiatric disorder, the Potocki-Lupski syndrome (PLS). Both SMS and PLS map to dysfunctions in the *Retinoic acid-induced 1* (*RAI1*) gene which functions through the RXR complex [15].

SHANK (SH3 multiple ankyrin repeat domain protein) is associated with ASD through its interactions with synaptic proteins as a scaffolding complex. SHANK belongs to a class of protein complexes of unique subunit/subdomain construction which are cytoplasmically expressed and then posttranslationally localized to regulate such diverse mechanisms as scaffolding, targeting, and autocrine signal transduction. SHANK provides a series of protein assemblages that orchestrate multiple protein interactions [16]. 

To understand the role of SHANK family proteins, the interaction with other cellular proteins must be described. Certain postsynaptic density-95 (PSD-95) domain proteins help promote synaptic connections between neurons. A excitatory postsynaptic polypeptide called Densin-180 is scaffolded into its location via interactions with SHANK [17]. SHANK is a member of a larger protein family that is responsible for the organization of multiprotein complexes in the synapse. It was reported that SHANK also helps anchor the *β*PIX guanine nucleotide exchange factor for certain small GTPases. SHANK is found in association with *β*PIX at excitatory synapses. The SHANK protein facilitates *β*PIX signaling which includes a p21-associated kinase (PAK). SHANK causes synaptic accumulation of *β*PIX and PAK, thus providing key insight into the role of this scaffolding protein for excitatory postsynaptic mechanisms [18]. SHANK is also directly implicated in glutamate receptor-mediated neuronal signaling. SHANK is involved in the scaffolding of glutamate receptor polypeptides along with the postactivation signal transduction complex and associated cytoskeletal proteins of the “postsynaptic density” [19]. Because of its association with the glutamate receptor, SHANK also participates in scaffolding the release of Ca^+2^ ions from the smooth ER triggered by neuroligin, ErbB4, and NMDAR [20]. SHANK is evolutionarily linked and cellularly associated with the SYNTENIN protein family as both share a PDZ protein-protein interaction domain. SYNTENIN is involved in neuroimmunobiological processes discussed below [21, 22].

ASD and tuberous sclerosis complex (TSC) are comorbid in children. TSC is found in only a few percent of ASD, whereas ASD symptoms are found in up to 50% of children with TSC. TSC presents with seizures and cognitive impairment, and these disorders share genes that may be related [23]. The genes implicated in TSC include the *β*-catenin/WNT-2 gene family. Besides these genes, the TSC 1 and 2 genes are directly linked to the disorder and therefore may be considered as risk factors for ASD. Like ASD, the tuberous sclerosis complex is a neurogenetic disease characterized by loss of function of certain genes. If either TSC1 or TSC2 are lost, severe symptoms such as mental retardation and epilepsy are found. TSC1 and 2 form a complex which controls the mTOR (mammalian target of rapamycin) which is an SER/THR kinase known to regulate transcription and cell cycle. Neurons which have lost expression of the TSC1/TSC2 complex are prone to apoptosis [24]. Tuberosis sclerosis, like autism, is a complex disorder that may be considered a member of the ASD family. It is established that the TSC1/TSC2 gene products control mTOR, and they are implicated in the ß-catenin/WNT pathway known to control neuronal transcription [25].

 The function of *β*-Catenin is to control cell adhesion along with the cadherins. *β*-Catenin is also involved in the downstream processing of the WNT transcriptional pathway. In this capacity, the inducible nitric oxide synthase (*hiNOS*) is controlled by the the WNT/*β*-catenin pathway. Indeed, the WNT/*β*-catenin pathway regulates the cytokine- and tumor necrosis factor *α* (TNF-*α*)-induced hiNOS expression via NF-*κ*B [26]. Cytokines such as interleukins as well as the TNF-*α* have been implicated in ASD (see below). 

Certain proteases play a critical role in mammalian development as well as in the immune response. The glycoprotein Reelin is a glycosylated serine protease that plays both developmental and house-keeping roles in neural development and cognitive function. Reelin is involved in the concerted migration of both neural and glial cells during mammalian brain development in the embryo [27]. This protein is also important in neurotransmission. Alterations of the Reelin pathway via mutation deletion or amplification as well as epigenetic alterations of the Reelin promoter region have been implicated in several neuropsychiatric illnesses including autism. The Reelin polypeptide is composed of several domains, each of which functions in some aspect of biological activity including specificity and membrane localization. The CR-50 subdomain is necessary for Reelin oligomerization. Mutations in the CR-50 region completely destroy Reelin biological activity. Reelin binding ultimately leads to membrane-associated phosphorylation cascades that facilitate GABAergic neurons [27].

Reelin is involved in global CNS development and maturation, and ADS is a disorder that appears to be derived from mistakes along the developmental axis. Indeed, there have been indications in ASD brains for anatomical malformation in the cerebellum, hippocampus, and the frontal and parietal cortex. These general observations could provide a link between Reelin biological activity and autism. Indeed, Reelin activity has been shown to be greatly diminished in ASD children. Reelin polymorphisms involving trinucleotide repeats are associated with autism [28]. Since Reelin is necessary for neuronal development and cellular migration, its activity in immune responses that require cell adhesion and receptor-mediated autoactivation may be relevant in ASD.

While serotonin is widely recognized as a neurotransmitter involved in several neuropsychiatric disorders, its role in ADS has been less obvious. However, work in rodents has suggested a correlative role for serotonin levels and brain development. Inhibition of serotonin receptor binding in gestating rats leads to major anatomical abnormalities in fetal and neonate brain development [11].

In humans, the level of serum plasma serotonin in both autistic mothers and offspring was lower than in normal mothers and children, while nonautistic fathers or siblings had serotonin levels comparable to normal subjects [29]. The serotonin transporter gene has gained some attention as being related to ASD. Increased levels of circulating serotonin have been reported in autistic patients. Since the serotonin transporter 5HT2A would remove free serotonin, the implication is that a reduction in transporter expression or availability could be correlated to ASD. Single-nucleotide polymorphisms (SNPs) in human chromosome 13q where the 5HT2A resides have been reported for ASD. To examine the possible link between the serotonin transporter and autism, several nucleotide polymorphisms were mapped and haplotype transmission was scored. These included an SNP in the proximal promoter region of the gene (−1438 G/A), a second SNP at nucleotide 102 (T/C SNP) and a third SNP located at intron 1 directly proximal to exon 2. A fourth SNP within exon 3 that induces a nonconservative amino acid change (His452Tyr) has also been examined [30]. Because of the small sample size of this study, it was unclear how the mutations were inherited or if they arose *de novo*.

However, since several of these mutations map to the serotonin transporter gene and hyperserotonemia is common in certain forms of ASD, a link between this neurotransmitter and ASD has been suggested. A recent report suggests that there is a correlation between the level of the cortical 5-HT 2A receptor and some cases of Asperger's syndrome [31]. The authors of this report were quick to point out that their study included only a small adult sample, and any suggestion that juvenile Aspergers or ASD in general are caused by a reduction in serotonin transporter expression was as yet not established. The authors go on to advise that a reduction in the expression of 5-HT2A in several regions of the brain including the frontal and temporal cortex appeared to be associated with diagnostic criteria for ASD including poor or withdrawn social engagement. Since the cortex is involved in social language and communication skills, there may still be a correlation between serotonin transporter expression and these ASD diagnostic criteria. 

Within the diagnostic criterion for ASD, there are some specific syndromes that should be included although they might diverge from the bulk of clinical presentations. Rett syndrome (RTT), while decidedly within the ASD diagnosis, affects female rather than male children at onset. Rett syndrome is an extreme neuropsychological disorder that appears in early female development and involves physical abnormalities as well as cognitive and behavioral deficits. Rett falls under the ASD paradigm as a neurodevelopmental/progressive debilitation. Early neo- and postnatal growth are normal and unremarkable, but this progression is followed by an interruption in growth along with loss of directed hand movement. Eventually head and brain growth become diminished along with cognitive abilities and normal movements such as sitting standing and walking (NINDS, http://www.ninds.nih.gov/). Unlike most ASDs in males, Rett is probably caused by only one genetic mutation within the methyl CpG-binding protein 2 (MeCP2) gene. The MeCP2 gene is located on the long (q) arm of the X chromosome in band 28 (“Xq28”), from nucleotide152,808,110 to 152,878,611. The *MeCP2* can regulate specific gene expression via epigenetic control over CpG islands in promoter regions of neuronal genes [32–34].

The MeCP2 protein binds to forms of chromatin-associated DNA that has been methylated via a promoter/enhancer-specific s-adenosyl MET transferase. The MeCP2 protein then interacts with other proteins to form a complex that turns off the gene. More than 200 mutations in the MeCP2 gene have been identified in females with Rett syndrome. These mutations include SNPs, insertions, and deletions. Since methylation control over gene expression becomes disrupted, some MeCP2-regulated genes may express inappropriately while others may be alternatively spliced due to CpG islands located at exon/intron junctions. These mutations appear to severely alter brain development. In a paper that describes how mutations in the MeCP2 can effect DNA/protein interactions and thus disrupt control over epigenetically regulated neural genes causing Rett, the following SNPs were examined: R106W, R133C, F155S, and T158M [35, 36]. These mutations are found in the MeCP2 methyl-DNA-binding domain (MBD). It was determined that each mutation played a significant role in the stability and folding dynamics of the MeCP2 polypeptide, thus rendering it relatively inactive. Some of these mutations could map onto varying severity of Rett syndrome presentation. This result points to the complexity of ASD genetic analyses.

 A recent study examined Rett and coincident MeCP2 mutations in a small Tunisian cohort [37]. They demonstrated the co-occurrence of a double MeCP2 mutation in a single patient: R306C and 1461+98insA. This mutation may cause a new polyadenylation site in the untranscribed terminal region of the gene thus altering transcript size. They also found a second MeCP2 polymorphism in the 3′UTR upstream from the polyadenylation site. Besides these two new mutations, 2 common mutations were spread among the remaining five females in the study. This suggests that several SNPs can be found within a relatively small portion of the genome, and each of those reported will both disrupt normal expression of the MeCP2 polypeptide and present with Rett syndrome. 

While Rett syndrome is almost certainly associated with mutations in MeCP2 which corresponds to the epigenetic protein machinery of DNA methylation, ASD in general may have more complicated and therefore varied biological origins. However, unlike other forms of ASD where multiple genes and/or large deletions and additions have been documented, specific mutations in the *MeCP2 *are found consistently in female infantile autism as well as in pronounced neonatal encephalopathy.

The mutations in MeCP2 may help to explain how distantly linked genes and loci can contribute to ASD. Autism often maps to the noncoding regions of the human genome. Besides copy number variation and insertional inactivation mechanisms, these regions may also directly control gene expression via epigenetically programmed methylome maintenance. These nonprotein coding genomic regions sometimes cohere with promoter and enhancer regions, necessary for RNA polymerase binding. 

MeCP2 expression is temporally and spatially regulated during gestational and infantile development. A closely linked upstream gene on the X chromosome named cyclin-dependent kinase-like 5 (CDKL5) may also be involved in Rett syndrome [38]. These observations help to illustrate the complexity of ASD. Namely, corruptions in developmental physiology and genetics via mutations in specific chromosomal loci may lead to subsequent biochemical modifications of chromatin that results in the incorrect timing of critical gene expression patterns. This is clearly the case with the MeCP2 gene and could explain the effects of other signaling pathway gene products such as CDKL5. 

Besides direct protein-level mutations leading to misfolding, poor ligand binding, and increase in either stability or degradation, the inappropriate timing or tissue distribution for gene expression could lead to a corruption in brain development. It is likely that both phenomena could interact at an epigenetic level via gene products like MeCP2, thus elaborating a molecular prerequisite for neuropathological endophenotypes. A corruption in behavioral phenotypes would occur if these molecular changes in the brain and CNS modified basal developmental programs. 

Since MeCP2 expression can directly modify brain development, a study was conducted to examine the heterogeneity of expression in various neuronal preparations. The gene appears to be active in all cell subtypes, but there are differences in the level of expression. Using a frontal cortex-derived neuronal microarray generated from ASD patients and age-matched normal patients, a quantitative experiment was conducted [39]. Two forms of MeCP2 were detected, each exhibiting a difference in the overall level of expression. Cerebral tissue from normal subjects had a steady increase in MeCP2 transcription over time. Cerebral tissue from diseased patients was strikingly different at both the transcriptional and posttranscriptional levels, suggesting that diverse molecular mechanisms contribute to the complexity of MeCP2 expression found in ASD. 

MeCP2 regulates the developmental program in GABAergic neurons. In a study of excitatory neurons of the ventrobasal complex (VB) of the thalamus and inhibitory neurons of the reticular thalamic nucleus (RTN) in mice, a deletion of MeCP2 decreased GABA response in VB neurons and had a reciprocal effect on RTN neurons. However, no differences in paired-pulse ratios of evoked GABAergic responses were found between MeCP2 knockout mice and controls [40]. Paired-pulse depression is an indication of short-term synaptic plasticity. Antibodies were used to stain for vesicular GABA transporter, and it was determined that there was a decrease in GABAergic synaptic terminals in *MeCP2* knockout mice. Therefore, this study suggests that MeCP2 is involved in the development and regulation of thalamic GABA signaling in both excitatory and inhibitory neurons, but that synaptic plasticity as measured by paired pulse depression was spared [40].

The anatomical and tissue-specific changes in Rett syndrome may be of direct consequence to a deficiency in MeCP2. There is a decrease in dendritic arborization in RTT, and recently, clues were found that link the MeCP2 levels to this anatomical abnormality. This group conducted analysis of cytoskeleton-protein expression in ASD brains versus control brains. Both TUBA1*β* and TUBA3 (*α*-tubulin genes expressed in neurons) were greatly underexpressed. A decrease in the level of *α*-tubulin is linked to abnormal cell biology. After transformation of these cells with a native copy of the human MeCP2 gene, cell morphology was restored to normal [41]. This could point to a possible strategy for repairing the null MeCP2-regulated cellular abnormalities found in ASD.

A review of ASD would not be complete without a description of the fragile X syndrome. We have already discussed a few genes that are found on the X chromosome. This discussion is for a specific set of mutations found only on the X chromosome in humans that have been repeatedly implicated in autism and many forms of mental retardation. Fragile X syndrome is a progressive/developmental disease that involves a complex set of cognitive, learning, and behavioural abnormalities. As with most ASDs, fragile X tends to occur more often in males who also experience the more severe symptoms.

Individuals with fragile X syndrome often present with hyperactivity, anxiety, impulsivity, spontaneous movements, and excessive fidgeting. Sometimes fragile X patients may be diagnosed as attention-deficit disorder. A large proportion of males with fragile X syndrome would be diagnosed with autism or more generally ASD. Fragile X is also characterized with seizures and epileptic episodes [42].

The physical manifestations of fragile X become increasingly abnormal with age especially in males. These physical characteristics include an extended narrow face, oversized ears, jutting and prominent jaw and forehead, and sometimes macroorchidism, especially after puberty [42].

Nearly all cases of fragile X syndrome are caused by a mutation in which a DNA segment known as the CGG triplet repeat is expanded within the FMR1 gene. Normally, this DNA segment is repeated from 5 to about 40 times. In people with fragile X syndrome, however, the CGG segment is repeated more than 200 times. The abnormally expanded CGG segment turns off (silences) the FMR1 gene, which prevents the gene from producing fragile X mental retardation 1 protein. Loss or a shortage (deficiency) of this protein disrupts nervous system functions and leads to the signs and symptoms of fragile X syndrome [43].

 The fragile X mental retardation protein (FMRP) is the most important candidate gene for the syndrome. FMRP is an RNA-binding protein that plays a role in the control of synaptic gene expression. In a recent paper, FMRP knockout mice were examined for the expression of both neurotransmitter receptors and a set of scaffolding proteins (including SHANK) necessary for their biological activity [43]. In FMRP knockout mice, both scaffold proteins and neurotransmitter receptor subunit expression in postsynaptic densities (PSDs) are modified. Both in the neocortex and hippocampus, SHANK1 and SAPAP scaffold proteins and various glutamate receptor subunits are affected. FMRP expression appears to alter translation or posttranslational machinery rather than transcription since transcript levels of affected genes are somewhat constant in knockout versus normal mice, but protein levels are increased suggesting that the normal function of FMRP is to suppress the translation of the scaffold protein glutamate receptor complex. Since FMRP normally suppresses SHANK protein levels, the loss of this gene in fragile X would promote the activity of SHANK-induced dendritic spine maturation leading to the disease symptoms. 

In a related paper, FMRP was shown to regulate translation of dopamine neurotransmitter-associated transcripts in dendritic spines [44]. The neurotransmitter dopamine (DA) is implicated in a host of neuropsychiatric conditions when alterations in its activity, availability, and receptor and transporter expression have been observed. Besides its direct role(s), DA is also implicated in the control of synaptic plasticity. Whether this mechanism plays a role in FMRP-mediated ASD is not established. In a study with a small cohort of children with ASD, dopamine transporter levels were not different from a control group [45].

It is known that DA binding to its receptor modulates brain function through pathways that are responsive to protein synthesis inhibitors. It was shown that FMRP is important for the dopaminergic receptor-controlled expression of synapse-associated protein 90/PSD-95-associated protein 3 (SAPAP3) in the prefrontal cortex (PFC). It appears that DA receptor stimulation involved reversible induced phosphorylation of FMRP. Indeed, phosphorylation of FMRP was closely followed by the DA receptor-induced accumulation of SAPAP3. SI RNA-mediated knockdown of SAPAP3 inhibited PFC neuronal internalization of GluR1. All of this suggests that FMRP may regulate glutamate receptor internalization via a DA-mediated pathway [44].

Besides the direct effect of fMRP on the Fragile X syndrome in ASD, other factors have been implicated. Since FMRP regulates RNA stability, several downstream gene products can be affected by one global mutation. Indeed, because FMRP downregulates expression at the translational level, entire pathways or circuits in the brain may be altered simultaneously in the CNS of ASD patients during development. Some obvious phenotypes observed in FMRP knockout mice include severe cognitive and behavioral malfunctions as well as endophenotypic alterations in the glutamate receptor. The previous papers examined some of the effects of FMRP on scaffolding proteins and neurotransmitter receptor subunit expression which partially explain this phenotype. Another recent paper implicates the mTOR pathway [46]. mTOR or mammalian target of rapamycin is controlled by the glutamate receptor cascade and has been shown to regulate neuronal translational machinery. In this recent paper, it was demonstrated that mTOR phosphorylation (which amplifies activity) was increased in FMRP knockout mouse hippocampus. It was suggested that mTOR signal increase (as allowed by the loss of FMRP) could cause enhanced activity of the glutamate receptor which in turn could alter synaptic plasticity. Alteration of plasticity in developing (or even mature) synapses may have long-reaching effects on action potentials and ultimately cognitive and behavioral patterns in ASD patients.

Besides these canonical genes and molecular genetic systems, a number of new genes have been described as potential candidates for ASD. Since ASD is a collection of autistic disorders, there should be no surprise that multiple genes and gene families would be unearthed during the mining of the genome for causes to autism. In the next section, we will briefly summarize some of the new genes, molecules, networks, and pathways that appear to be linked in a systems-wide emergent story that holds hope for understanding and eventually treating ASD in our children. The genome-wide mechanisms for mutation which include copy number and sequence variation and epigenetics will be mapped onto this literature along with an appreciation for the global contribution of the immune response and genetic recombination/repair.

## 3. ASD Genomics

In modern genomics since the complete sequencing of the human genome, many new techniques and software technologies have arisen to better examine large libraries of data. Using these new techniques (array comparative genomic hybridization *aCGH*, high-density SNP genotyping platforms, and deep sequencing), many new genes have been identified as candidates for causing or at least correlating with ASD. To deal with this massive amount of data, these new technologies have themselves opened up new ways to look at biological systems especially those that can be dissected at the genomic level. Unlike many other human diseases, autism can be visualized microscopically. Establishing structural motifs in chromosomal abnormalities have suggested that such features can occur relatively frequently [47]. Upon examination, it has been shown that duplication in the maternal genome at chromosome 15 can account for up to 3% of all reported ASD cases [48]. Duplications can give rise to copy number variations (CNVs) which have become the new paradigm in understanding complex traits and diseases such as autism. This seems to be a common mechanism for neuropsychiatric diseases in that duplications and deletions have been associated in CNVs for genes involved in neural development and synaptic plasticity in ASD, schizophrenia, and general mental retardation [49].

There is great heterogeneity in all genomes, and this variation in specific sequence tends to increase with an increase in nonexpressed DNA. Even though mere 20 to 25 K genes actually comprise the functional (read transcribed) genome in higher primates, including man, more than 95% of the genome is not used for that purpose. It is these regions of the genome where allowance of mutations is increased. After the sequencing of the human genome and after years of careful molecular genetic studies on health, development, disease, and death, it has become clear that this vast untranscribed region of the genome plays a very essential yet still quite intractable role in human biology. What this suggests is that a standardized text-book reference genome for all of human biology is not an appropriate concept. Indeed, the devil is in the details. There are a variety of classical genetic mutations where unique nucleotides have been substituted or deleted or added to a fixed sequence both within genes and in the vast region that is not transcribed. Besides these classical single-nucleotide polymorphisms, there are triplet repeats that can themselves be reiterated hundreds of times (as in fragile X) as well as small-scale to large-scale (megabase) insertions and deletions. Deep sequencing of the human genome has revealed many millions of SNPs with smaller distributions of repeats, insertions, and deletions. There are also frame-shift mutations and gene translocations which can cause aberrant gene expression and sometimes disease. Indeed, all mutations can lead to disease. While some mutations are inherited and some arise *de novo*, importantly in development and disease of development like ASD, both of these mechanisms occur. Without a complete sequence of each individual, there will always be a lack of knowledge as to the potential for mutation-associated disease. Besides cancers, which often arise from genetic disposition followed by environmental stimulation, disease of the central nervous system can be characterized in a similar way. However, unlike cancers, disorders such as ASD may have many (hundreds, perhaps thousands) unlinked genetic predispositions and many cues from the environment to trigger the wayward development of the CNS during gestation, early infancy, and childhood. 

There are several reports that contribute new information regarding the importance of copy number variation and autism. Using a comparative genomic hybridization screen (CGH), it was shown that *de novo* CNVs were isolated in heterogenous genomic regions in ASD patients at frequencies which reached 10% as compared to 3% in patients who had a first-degree relative with ASD and only 1% in normal controls. While these numbers are not large, remember that these are very rare mutations and as such represent a significant risk factor for autism in these populations. Several gene candidates were isolated in this study. One that may merit further study is the FLJ16237 gene which codes for a stearoyl CoA desaturase enzyme which has been detected in the superior temporal gyrus of fetal brain [50]. Stearoyl CoA desaturase is a critical enzyme necessary to produce oleic acid, the critical monoenoic 18-carbon fatty acid found in sphingo- and glycerolipids throughout the CNS and in particular in association with the blood brain barrier [51].

Other reports on CNVs and autism include a *de novo* deletion in chromosome 1p34.2 which mapped to the regulating synaptic membrane exocytosis 3 (RIMS3) [52]. RIMS3 had previously been reported as a potential gene involved in schizophrenia where it is expressed in the cytoactive matrix zone of the amygdala [53].

It has been shown that microdeletions in chromosome 16p11.2 have generated copy number variants that are found in a small but constant number of ASD cases [54–56]. This single microdeletion CNV represents the second most common chromosomal disruption associated with autism and duplication of this region (~500 kb) has also been implicated in ASD [57, 58]. Besides chromosome 16, the highest proportion of autism-linked maternal chromosomal abnormalities is found on 15q with other less frequent deletions or duplications on chromosomes 2q and 22q [47, 59].

Using modern bioinformatics and microarray technology, a new gene in chromosome 16p11.2 has been described which could be an important candidate for ASD. These researchers found a coding variation in the *SEZ6L2* gene (R386H) which covaried with autism (*P* = .014) [56]. The *SEZ6L2* is an ortholog to a mouse gene that is linked to seizures, and since ASD has high incidence of epilepsy, the human variant is a likely candidate for the cause of this epilepsy. The mutation maps to a region of the protein critical for axonal growth which further suggests a correlation to the epileptic component of ASD. Another report from the same lab has uncovered a CNV on chromosome 15q 13.1 that codes for neuronal adaptor protein, the amyloid precursor protein-binding protein (APBA2) [60]. In this instance, the mutations in this gene involved copy number and sequence variation both of which appeared to be inherited from either the mother or the father in independent siblings with autism.

In a broad-based CNV study examining ASD susceptibility genes, a glycobiological network emerged as contributing to the disease [61]. Since global genomic perturbations seem to play a role in complex biological systems such as developmentally linked disease, the next logical movement is to find connections among these diverse polymorphisms. To account for the complexity of the system and relationship of CNVs to either duplication or deletions or simple SNP variation within loci, biotechnology has generated software to analyze the data. Once this data has been accumulated for ASD cohorts with specific histories and clinical presentations, it is possible to start organizing genetic parameters around an endophenotypic network such as a metabolic pathway or cellular system. These researchers used a gene-network reconstruction designed for the human genome called Prioritizer. Using this approach, clusters and networks of genes were assembled which organized around a neurobiological motif [61]. The following is a battery of ASD susceptibility genes. These include *Retinoic acid-induced 1* (*RAI1*), from chromosome 17p11.2, which has already been implicated in cognitive and behavioral insufficiencies, *Bromodomain-containing protein 1* (*BRD1*) from chromosome 22q13, that has been linked to schizophrenia and bipolar disorder, and the *LARGE* gene, on chromosome 22q12.3, which has been implicated in mental retardation and schizophrenia. 

The LARGE gene product is required for the efficient glycosylation of a cell surface receptor protein for arena viruses including the lymphocytic choriomeningitis virus (LCMV) which is the human pathogenic Lassa fever virus. Therefore, LARGE serves as a required factor in the virulence of a pathogenic virus and is generally necessary for host-cell-viral interactions [62]. LARGE is also critical for producing the correct glycosylation of both *α* and *β* distroglycan receptor via its activation of a acetylglucosaminyltransferase that promotes tumor suppression. Thus, another physiological role for LARGE is to correctly glycosylate surface receptors to block tumor metastasis [63].

RAI1 has been shown to mediate early antiviral response and toll-like receptor (TLR) 3 expression, thus playing a neuroimmuno protective role [64]. RAI1 is also a component of the innate immune response where it is involved in causing an increase in interferon [65]. Finally, RAI1 is also implicated as a signaling molecule (as Shc) involved in cell proliferation, differentiation, survival motility, and migration [66]. Besides these, other genes that have key roles in the development and operation of the CNS were isolated. These include neurotrimin (NTM), piccolo (PCLO), D4 zinc and double PHD fingers family 1 (DPF1, also calledNeuD4), and S100 calcium binding protein A5 (S100A5). These gene products have all been localized to important brain regions known to be affected in autism including the cerebral cortex and hippocampus. 

Most interesting from this gene network analysis were the cluster of glycobiological genes that mapped to ASD according to CNV. It has been known for some time that glycosphingolipidoses are common in severely retarded children. These inborn errors of metabolism are often classed as membrane glycolipid and glycoprotein storage diseases [67]. Since these disorders have been thoroughly described in the literature, it may seem that no new information has been obtained by using the genomic network strategy. However, the new facts that arise from this paper are most helpful in understanding the ASD paradigm since CNV of these genes points to effects of gene dosage rather than null alleles. The authors further point out that gene dosage could also explain how some ASD is sex-linked since a few of the CNVs found in their study arose *de novo* in the male subjects. This *de novo* increase in CNV of disease genes as a result of deletions or duplications of chromosomal segments can be caused by mechanisms of recombination, replication, and exon shuffling [61]. Of the CNVs involved in glycobiology, three were *de novo* and 3 others were from asymptomatic heterozygous mothers [61]. Both the spontaneous nature of *de novo* CNV and the inheritance from one parent could account for incomplete penetrance of the more severe pathologies typically found in glycosphingolipidoses. 

Finally, all seven CNVs found in the glycobiological network have been implicated in CNS disease in both humans and in animal models. Some of these gene products are involved in O-glycosylation, while others are in the protein-linked N-glycosylation pathways. In the present study, genomic losses and gains in genes encoding enzymes involved in all of these glycosylation pathways are apparent. The genes found were either deleted or duplicated and included the following: *LARGE*, *GALNT5*, and *GALNT9*, all of which are involved in O-glycosylation. *B3GALT6*, *B4GALT1*, and *GCNT2* are all genes whose products are enzymes controlling N-protein glycosylation. Many neural proteins including those which act as receptors and some of which are secreted share N-linked glycosylation as a posttranslational modification necessary for biological activity and correct cellular and temporal expression. The last of the glycobiological genes that were picked up in this CNV screen was *ARSA* that codes for a lysosomal arylsulfatase A which is essential for cerebroside metabolism [61].

As time goes on, these new technologies for genomic mining of CNVs and complex networks of genes will uncover the endophenotypes responsible for ASD. In the last section of this paper, we will take a look at some systems biological approaches in dissecting the causes and underlying pathologies of ASD that may allow us to more completely understand the interaction between susceptibility genes, genomic rearrangements, and the environment.

## 4. Systems Biology of ASD

Now that we have examined some of the principle genes involved in ASD and the genomics of their characterization, we will now briefly turn our attention to some new research that may lead to an ultimate comprehension of the disease. Starting with genes and gene products and looking at genomic rearrangements and mutations does not allow us to assume that we have gotten very far in understanding the causes of autism. The genes provide an initial gateway, but clearly one has to also grasp predisposition (e.g., maleness), various difficult to study epistatic interactions, and of course critical environmental factors, including antigenic, infectious, nutritional, and social ones that can modify developmental landscapes to promote autistic symptoms. In order to do that, perhaps we need to look at the overall biology of the system to better situate the autistic child in the environments where this developmental disorder has its multifactorial origins. Indeed, because of these complexities, and the desperate need for new and effective treatments, sometimes it may be most important to fathom the final-common pathways of brain-mind-body functions that lead to autism. That, along with genetic animal models of autism, will be the final consideration we will address in this paper. 

### 4.1. The Neuroimmune Connection

Several years ago a landmark review was written which included a great deal of work suggesting that the immune system may be involved in the onset and outcome of ASD. In this paper, it was emphasized that an usual paradigm for immune chemistry was observed in some cohorts of autistic children. These consisted of unusual ratios of lymphocytic (Th1/Th2) cells and numbers as well as an unusual abundance or deficit of certain cytokines, immunoglobulins, and complement [68]. Specifically, the authors summarized in a table the following: ASD patients had a decreased level of naïve T cells, an elevated number of circulating monocytes macrophages, reduced natural killer cell activity, and changes in proinflammatory interleukins and TNF-*α*. These early reports on the alteration of immune biochemistry and cell response suggested that alterations in neuroimmune pathways were evident in ASD. These changes can be induced by antigen and/or pathogen attack but if inherited and penetrant in a disease complex may suggest genetic control and perhaps a similarity to autoimmune disease. Genetic interactions are certainly necessary to produce a functional immune system, and aberrations in both innate and acquired immunity have increasingly become reported in a vast array of neuropsychiatric illnesses, some of which carry heritable components. 

ASD is a neurodegenerative psychological disorder of the brain. Immunological investigations as well as those associated with SNPs, CNVs gene expression, signaling, translational control, and anatomical abnormalities are almost always conducted on the neural tissue. The neurons of course are connected via axons and dendritic spines through synapses of which all brain information appears to flow. Therefore, a disease which alters so many brain functions including cognition, behavior, learning and memory, and motor skills is believed to arise from dysfunctions in neurons and synapses. However, the brain is largely composed of glial cells which also signal, though not via the conductance of action potentials. Besides being involved in communication, the glia are also essential as a cellular blueprint for the wiring of the brain by generating the platform through which the neural tissue grows and migrates through innervation pathways. The glia guide neuronal growth migration and adhesion by producing molecules that facilitate this process. A subset of glia, the microglia, are the brain's immune cell system. Cytokines, chemokines, and all other forms of the immune response are provided in the brain by the microglia. Other glia, the astrocytes, are involved in forming the blood brain barrier, which maintains strict control over the movement of cells, disease causing agents, and bioexogenous and endogenous molecules [69]. Glia have been implicated in neurodegeneration, a hallmark of ASD. Brain development is a genetically programmed process that is immunologically and epigenetically sculpted through interaction of the senses and the biochemical/biophysical environment. Perturbations in this process will necessarily modify brain functioning and if divergent enough could result in diseases such as ASD.

A recent paper examined the role of glial cells in the suppression of neurodegeneration which relates to this discussion. It has been established that activation of microglia can induce neuroprotection during inflammation in the CNS [70]. To examine this theory, a selective inhibitor of proinflammatory cytokines was administered to mouse brain expressing the human amyloid *β* (A*β*) protein. Hippocampal A*β* has been shown to induce IL-1*β*, TNF-*α*, and S100B, all proinflammatory cytokines. Administration of the permeant drug (4,6 diphenyl-3-(4-pyrimidin-2-yl) piperazin-1-yl pyridazine) suppressed neuroinflammation and restored hippocampal neurotoxic peptide levels to normal. This neurochemical recovery was accompanied by restoration of Y maze behavior suggesting behavioral normalization in the mouse. This study shows that microglia play a decisive role in neuroprotection via control over proinflammatory cytokine production in situ. Similar results were observed in an Alzheimer's animal model suggesting a persistent role for microglia in neuroprotection of brain disease [71].

A recent paper has taken a look at the possibility of infection playing a role in ASD. There is a suggestion that some of the cognitive and behavioral presentations of ASD could be linked to viral-mediated production of the proinflammatory cytokine interleukin 1*β* (IL-1*β*) [72]. Here, they used immunocytological methods to measure peripheral blood (PB) monocytes in an ASD cohort frequently infected by viruses consisting of 14 subjects (median age 7.8 yr) and compared them with 11 age comparable ASD control children (median age 6.8 yr) and 5 normal children (median age 8 yr). Both the ASD groups were ones that had regressed from early development into autistic cognitive and behavioral decline and who had developed certain dietary allergies. The virally challenged ASD test group had lower IL-10 production after challenge with known antigens as compared to normal controls. They produced an RNA microarray of all the subjects to examine differences in mRNA transcript levels. Their results showed an increase in some 1593 transcripts in the ASD test subjects and some 2704 transcripts in the ASD controls as compared to normal controls. They also measured specific IL-1 inducible genes and determined that some of them were higher in the ASD test group (*P* = .002). The cytokines IL-2, IL-3, IL-9, *α*-integrin, and TNF-*α* pathway transcripts were higher in the ASD control group. After screening over 800 common transcripts in both ASD groups, it was determined that IL-2 and IL-3 pathway-related genes were preferentially expressed. In order to interpret these results and to ascertain what they might suggest for the idea of viral infection involvement, it is necessary to know something about cytokine metabolism. The Interleukin 1 family of cytokines is generated from the myeloid cell lineage and as such is produced by macrophage monocytes, fibroblasts, and dendritic cells. IL-1*β* is a proinflammatory cytokine involved in cell adhesion by inducing endothelial cells to arrest the migration of circulating leucocytes and for recruitment of lymphocytes. Furthermore, IL-1B is involved in the differentiation and apoptosis of immune and infected cells. IL-1*β* is also responsible for inflammatory hypersensitivity where it activates the cyclooxygenase pathway generating prostaglandins and thromboxanes. Besides these important proinflammatory effects, IL-1B polymorphism is associated with the production of IL-2 in lymphocytes. Interleukin 1*β* is of a superfamily of interleukins which also include the IL-RA receptor antagonists [73]. Therefore, viral infection-induced increases in IL-1*β* family of cytokines could suggest a correlation between infection (or inflammation) and ASD since control children did not react, in a followup report by the same group [74]. GI tract infection in ASD subjects was used to investigate cytokine-mediated inflammation. Using a cohort of ASD children (ASD test group), severe cognitive deficit apparently caused by infection and inflammation of the GI tract was used to examine transcript levels in peripheral blood monocytes. As in the previous experiment with viral infection, these children were compared to same-age ASD children without GI infection and to control (normal) children. The mothers of each group were also tested. The analysis showed that 568 transcripts increased, while only 72 were depressed in the GI-infected ASD test group versus 469 upregulated and 57 downregulated in ASD children without GI infection and to control (normal) children. The ASD control group had increased chemokine expression (CCL7 and CCL2) and decreased cytokine expression (IL-6). The ASD test group mothers had differential transcript levels as compared to ASD control (non-GI-infected) mothers. Finally, the GI tract-infected ASD test group showed a decreased accumulation of IL-6 and IL-1*β*. Also, most ASD test group children had higher levels of chemokine CCL7 and CCL2 than controls with a decrease in the expression of the inflammatory cytokines. The authors suggest that there is a differential effect of bacterial infection of the GI tract in ASD children and that this may correlate with neurodegeneration. It would be better to see if these distinct cytokine and chemokine patterns (e.g., putative neurodegenerative chemokines) are found during early stages of ASD presentation and if GI tract inflammation is linked to disease severity and ASD symptomatology later in life.

In a related paper, the effects of maternal cytokine production and bacterial infection were addressed as they related to fetal neuronal development. Since it is not fully understood how pregnant females ward off infection from agents like bacteria, there has been a perennial concern for fetal development. In this animal study, it was shown that IL-1 was responsible for a proinflammatory condition which would result in fetal neurodevelopmental dysfunction that was directly related to placental destruction by the immune system [75]. Using bacterial lipopolysaccharide (LPS) to trigger the innate immune response, placental apoptosis was initiated, and for fetuses that survived, there was a great diminishment of forebrain white matter as well as severe motor skill problems in the pups. To demonstrate that IL1 caused the morbidity and mortality along with inflammation and cell death, an IL1 antagonist protected against both placental and fetal CNS destruction.

Immunogenetic responses and malfunctions have been reported in other contexts of ASD. The X-linked interleukin 1 receptor accessory protein-like 1(ILRAPL1) is involved in the calcium-regulated release of vesicles containing neurotransmitters in the synapse. A mutation was mapped to this gene in autistic females [76]. In a study involving pregnant women, there is an increased risk for schizophrenia and autism via maternal immune activation (MIA). In animal studies, MIA can account for rat pup abnormalities in behavior, histology, and gene expression. It appears that IL-6 is the major cytokine involved in MIA [77]. MIA can account for some 14–21% schizophrenia in genetically susceptible offspring, and this is associated with increases in IL-6 and sometimes with detection of circulating antibodies to influenza virus. These studies reveal that the infection is not associated with the fetus but rather within the mother. In the animal study, both LPS and poly I : C can induce abnormal offspring suggesting that an innate immune response may be the defining mechanism of neural dysfunction. Both LPS and poly I : C increase maternal serum cytokine levels which can accumulate in amniotic fluid, cross the placental membrane, and enter into the rodent fetal brain. The bottom line is that IL-6 mediates MIA-induced aberration of fetal brain development, and significantly, IL-6 controls BDNF expression during gestation [78]. In a related study in pregnant women, those with either autoimmune disease (e.g., celiac, rheumatoid arthritis, IBD) or who become infected with influenza virus during pregnancy are 3 times more likely to give birth to autistic children [79]. Whether this is linked directly to MIA is not known. ASD and schizophrenia have been linked to aberrations on chromosome 6 which is densely populated with immune-system genes such as the major histocompatibility complex (MHC) [80–83]. MHC alleles (HLA, *human leucocyte antigen*, in humans) are the most polymorphic of all human genes, and they arise from one of the most gene-dense (and therefore most susceptible to deletions and duplications) regions of the genome. Class I MHCs are found on all cells and can present to cytotoxic T cells, while Class II MHCs are exclusively found on immune cells (macrophages and B cells) and other professional antigen presenting cells. MHC can resolve the activity of IL-1 and IL-6, both of which can cause fetal brain damage. The cytokine IL-2 induces cytotoxic activity in T cells but only if MHC is expressed. All of this immunochemistry points to complex feedback-regulated cellular and humoral mechanisms that function through HLA genes that are linked to ASD [84, 85].

If system biological mechanisms are playing a role in the genetics of ASD, it is clear that the immune response, as a fundamental physiological mechanism involved in development, defense, and disease, is involved. According to the hygiene hypothesis for the development of the immune system in early age humans, virus infection plays a major role from birth to 2 years of life. Compared to nonvirally infected children, T-lymphocyte production is increased while the cytokines IL4 and IL 10 decrease with age in control children [86]. IL-1 levels are not greatly affected by virus at this early age, but macrophages increase in number after viral challenge. In general, T cells increase with age and especially so with viral challenge, but macrophage levels are more of a product of viral infection. The immune system in infants is not well developed. Host responses to viral infection are typically mediated by Th-1 cytokines, and Th-2-derived cytokines inhibit the expansion of Th-1 from naïve T cells. Th-1 cells generate IL-2, IL-12, IFN4, and TNF-*α* while Th-2 cells produce IL-4 and IL-5. Interestingly, in infants, THh-2 cells dominate, and this may be the result of gestational maternal Th-2 domination. A diminished Th-1 induction (which can be the case in cleaner home and school environments) could delay or corrupt immune maturation in infants by favoring Th-2 dominance and the cytokines generated from that lineage. When this occurs, people carry allergies throughout adult life. It is the balance of the various cytokines which regulate the ratio of Th-1/Th-2 cells that shapes the immune response through life [86]. Alterations in this ratio were reported in ASD children [68].

### 4.2. Epigenetic Mechanisms (Dan, Not Sure Why the Following Is “Boxed” at Least on My Screen)

There is a rapidly expanding literature in the neurobiological field that points to epigenesis as a trigger for autism and related disorders. In a review written in 2006, it was mentioned that fragile X, MeCP2, and several duplications on chromosome 15q (e.g., UBE3-A, *GABRB3*) and 7q (e.g., the paternal genes *SGCE* (sarcoglycan epsilon) and a retrotransposon derived paternally expressed gene 10 (*PEG10*), both of which are apparent MeCP2-controlled genes) were known to involve methylation and were detected within maternally inherited regions of this chromosome [87]. In a recent review, this search continues, and several new candidate genes have been detected that are controlled by or that control the methylation state of the genome [88]. They report that deletions or methylation patterns on 15q likely cause Prader-Willi or Angelman syndrome, and this is dependent on whether the pattern is found on the paternal or maternal chromosome. However, most mutations in 15q were not attributable to DNA methylation patterns.

Another group sought to profile methylation patterns for genes potentially important is ASD. Here, they used a large-scale CpG methylation-profiling microarray analysis of a lymphoblastoid cell line from monozygotic twins who were discordant for ADS and their nonautistic siblings. They found differential methylation patterns between the discordant twins and between the twins and the nonautistic siblings. They uncovered genes that regulate transcription, neural development, apoptosis, and other physiological process often found dysregulated in ASD [89]. Both the retinoic acid-related orphan receptor *α* (*RORA*) and the apoptosis gene BCL-2 were methylated. They used immunohistochemistry to reveal depletion of the two proteins in the autistic brains. Since the normal function of BCL-2 is to suppress apoptosis, methylation at this locus as found in the ADS twins could serve to promote cell death, thus leading to neurodegeneration. The RORA gene product is a member of a nuclear receptor family that may be involved in cell differentiation, and perhaps more pertinent to ASD, RORA protein may be involved in arresting cell proliferation and progression through the cell cycle [90]. If the RORA protein is underexpressed via methylation dependent mechanisms, as in the ASD brain, presumably cell proliferation would be enhanced. This difference in two ASD-related methylation patterns thus produces opposite outcomes. In one instance, the methylation of BCL-2 would promote apoptosis, and on the other hand, the methylation of RORA would tend to promote cell proliferation. Of course whether the gene products play that role in this system has not been determined. The important point might be that epigenetic phenomenon plays a role in ADS in ways that have yet to be appropriately elucidated.

As a rule, epigenetic phenomena shape biological and disease activity. While the mammalian genome establishes the template for empirically discernable developmental and behavioural patterns, a more complex and variable sequence helps to produce the final phenotype. This latter—epigenetic phenomenon has increasingly become the subject of mammalian developmental biology and gene expression. This is nowhere more apparent when analyzing parental imprinting. Imprinting, like many neurological diseases including autism, demonstrates its effect along developmental and gender-associated pathways. The biochemistry of epigenetics involves several covalent modifications of nuclear chromatin as well as posttranscriptional gene silencing. Among these modifications are methylation of the C5 atom on cytosine residues found in CpG islands associated with promoter elements, methylation, acetylation, and ubiquitination and phosphorylation of cohering histones and the processing of double-stranded RNA in the generation of siRNA involved in gene silencing [91]. The mechanisms of these epigenetic phenomenon include the activities of methyltransferases, acetyl transferases, kinases, phosphatases, demethylases, deacetylases, E3 ubiquitin ligases (implicated in ASD), and RNase enzymes. The substrates for these reactions are either chromatin or in the case of the Rnase activities, double-stranded mRNA. S-adenosyl methionine (*SAM *or *Ado MET*) is the recognized nuclear methylation agent, deriving the methyl group from folic acid derivatives [92].

Acetyl CoA is used in acetylation of chromatin-associated histones in the process of chromatin remodeling which generally enhances gene expression downstream from ligand/receptor-mediated activation of the complex which may be in association with the nuclear ubiquitin/proteasomal pathways. Nuclear-associated posttranslational modifications of histone carboxy termini clearly alter chromatin structure and function. The major effect is a pronounced change in the physical-chemical accessibility of DNA-binding proteins to unwind the double helix and transcribe RNA. These covalent modifications are typically reversible, but they can lead to a complete removal of histones from the chromatin complex, thus inducing cell cycle constitutive gene expression. Indeed, while methylation tends to dissociate histones from the chromatin complex, demethylation tends to favor transcriptionally inactive chromatin. All of this modification can play a role in ASD since altered gene expression during development could lead down neurodegenerative pathways (e.g., BCL-2). Besides the specificity of the methyltransferases and acetyl transferases on certain histone polypeptides, there is also specificity at the amino acid sequence level. To generate changes in reactivity of chromatin to remodeling, only very specific covalently modified histone amino acid residues play a role. The discrete biochemistry of these epigenetic modifications is lysine methylation, acetylation, and ubiquitination, serine phosphorylation, and arginine methylation [93, 94]. 

The majority of these covalent modifications affect DNA accessibility to various proteins and they can alter protein-protein interactions among chromatin-bound histones and other polypeptides.

In a recent report, the 15q chromosome was again implicated as a site for epigenetic/methylation imprinting in ASD [95]. These researchers found that transcription rates were correlated with the dosage of methylation patterning in male autistic children presenting with cognitive deficit and spontaneous seizures. An autistic female also had significant deficiencies in several key genes that were paternally inherited (*SNRPN*, *NDN*, *HBII85*, and *HBII52*). The deficiencies in transcription of these genes were directly linked to DNA methylation although this individual had milder symptoms than the male children in this study. It was not determined if histone modifications were also present and this could play a role in long-term control over transcription rates of methylated genes. Considering the advances made in the role of DNA methylation in practically all bodily and brain functions, including learning and memory [96], the epigenetic analysis of all of the symptoms of ASD is bound to grow in the next few years. 

We have already discussed the MeCP2 gene and its substantial influence on ASD. It should be emphasized that mutation in this gene would have global consequences on methylation patterns across a spectrum of genes. What has yet to be determined is how *de novo* methylation is controlled and whether loss and gain of parental imprinting is substantially altered by environmental stimuli in autistic children. These studies wait to be performed against the backdrop of the increasing number of genes that are becoming implicated as susceptibility factors in this debilitating neurodevelopmental disease.

## 5. Conclusion

In this paper, I have attempted to catalogue a number of key genes implicated in the complex set of diseases called ASDs. Recombination and repair mechanisms play essential roles in the production of copy number variations that appear to be robustly responsible for many endophenotypic signatures in these disorders. 

**Figure 1:**
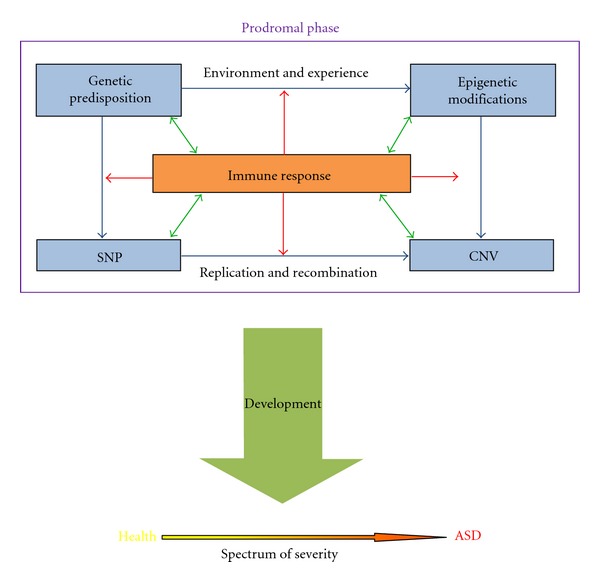
Hypothetical model linking genetic/genomic mutation to autism spectrum disorder through endogenous mechanisms of the immune response.

It is clear that autism is a profoundly complicated spectrum of disorders that have an underlying complexity of genetic components and genomic alterations that organize around a central theme of neural network infirmities and neuroimmunodysregulations.  Figure 1 shows how genetic and genomic predisposition may be linked via the immune response and development to ASD. Mechanisms of the acquired immune response use genetic recombination and rearrangement, clonal expansion, and cell death to eliminate exogenous (disease-causing organisms, xenobiotics, etc.) and endogenous (damaged and infected cells, tumors, etc.) pathogenic agents. These same mechanisms are responsible for copy number variation, mutation, and neurodevelopment, all impacted in ASD. Gene products which regulate nonhomologous recombination should be examined for their role in genesis of CNVs. It should be noted in closing that a strong comorbid condition of gastrointestinal disorders is quite common in ASD. That these disorders are related to autism is abundantly clear. What is less clear but could be speculated upon is the connection of immune or autoimmune mechanisms at work in both brain and GI tract dysfunction (i.e., gastrointestinal-associated lymphoid tissue, GALT). Besides this immune connection, the gut and brain share many neurotransmitters and neuropeptides with their accompanying receptors and signal transduction systems that modulate many basic social-emotional and other affective processes that are imbalanced in autism. In the future, it may become clear that ASD is a disease, in part, of the immune system as well as basic brain functions that regulate our emotional life.
